# Inhibition of PFKP in renal tubular epithelial cell restrains TGF-β induced glycolysis and renal fibrosis

**DOI:** 10.1038/s41419-023-06347-1

**Published:** 2023-12-12

**Authors:** Shu Yang, Han Wu, Yanchun Li, Lixin Li, Jiaqing Xiang, Lin Kang, Guangyan Yang, Zhen Liang

**Affiliations:** 1 Department of Geriatrics, The Second Clinical Medical College, Jinan University (Shenzhen People’s Hospital), Shenzhen, Guangdong China; 2 Guangdong Provincial Clinical Research Center for Geriatrics, Shenzhen Clinical Research Center for Geriatrics, The Second Clinical Medical College, Jinan University (Shenzhen People’s Hospital), Shenzhen, Guangdong China; 3 Department of Endocrinology, The Second Clinical Medical College, Jinan University (Shenzhen People’s Hospital), Shenzhen, Guangdong China; 4 The Biobank of National Innovation Center for Advanced Medical Devices, Shenzhen People’s Hospital, Shenzhen, Guangdong China

**Keywords:** Kidney diseases, Kidney

## Abstract

Metabolic reprogramming to glycolysis is closely associated with the development of chronic kidney disease (CKD). Although it has been reported that phosphofructokinase 1 (PFK) is a rate-limiting enzyme in glycolysis, the role of the platelet isoform of PFK (PFKP) in kidney fibrosis initiation and progression is as yet poorly understood. Here, we investigated whether PFKP could mediate the progression of kidney interstitial fibrosis by regulating glycolysis in proximal tubular epithelial cells (PTECs). We induced PFKP overexpression or knockdown in renal tubules via an adeno-associated virus (AAV) vector in the kidneys of mice following unilateral ureteral occlusion. Our results show that the dilated tubules, the area of interstitial fibrosis, and renal glycolysis were promoted by proximal tubule-specific overexpression of PFKP, and repressed by knockdown of PFKP. Furthermore, knockdown of PFKP expression restrained, while PFKP overexpression promoted TGF-β1-induced glycolysis in the human PTECs line. Mechanistically, Chip-qPCR revealed that TGF-β1 recruited the small mothers against decapentaplegic (SMAD) family member 3-SP1 complex to the *PFKP* promoter to enhance its expression. Treatment of mice with isorhamnetin notably ameliorated PTEC-elevated glycolysis and kidney fibrosis. Hence, our results suggest that PFKP mediates the progression of kidney interstitial fibrosis by regulating glycolysis in PTECs.

## Introduction

The incidence and prevalence of chronic kidney disease (CKD) are increasing annually worldwide. Diabetic kidney disease (DKD) is the most common cause of CKD and the main cause of end-stage renal disease (ESRD), posing a serious threat to patients’ lives [1, 2]. Although many achievements have been made in the treatment of CKD, therapeutic options for CKD are still limited, and constant satisfactory outcomes are not attained with the use of conventional approaches [3, 4]. Renal fibrosis, including glomerular sclerosis and renal tubulointerstitial fibrosis, is the main pathological change and a common outcome of many CKDs. Tubulointerstitium accounts for more than 90% of renal parenchyma and has various important functions. Emerging evidence suggests that renal tubular injury occurs earlier than glomerular damage and plays a substantial role in CKD progression [5, 6]. In addition, the development of tubulointerstitial fibrosis is a crucial predictor of CKD progression to ESRD. The mechanism of renal tubular injury mediated by CKD is complex and involves metabolic abnormalities, hemodynamic effects, and inflammatory responses [7].

The kidney is one of the organs with the highest metabolic rates, which is mainly driven by renal tubular epithelial cells (TECs). The kidney needs to maintain energy homeostasis, and abnormal energy metabolism can lead to cell dysfunction, cell death, and a wide range of kidney diseases [8]. Under normal physiological conditions, proximal TECs (PTECs) rely almost entirely on mitochondrial fatty acid oxidation (FAO) to generate energy, which consumes a large amount of molecular oxygen [9]. After kidney injury, the FAO ability of PTECs is impaired, leading to metabolic reprogramming of cells to provide energy [10]. Metabolic reprogramming, a shift from mitochondrial FAO to glycolysis in PETCs, is considered to play an important role in CKD progression [11]. In the case of diabetes, due to changes in metabolic substrates and oxygen delivery, renal tubules suffer from hypoxia, glycolysis, and lipid accumulation, resulting in increased production of reactive oxygen species (ROS), proinflammatory and profibrotic factors, and increased PTECs apoptosis and renal fibrosis [11, 12]. Therefore, improving energy metabolism is a new strategy for CKD prevention and treatment.

Many studies have shown that glycolytic enzyme expression is altered in patients with diabetes [13]. Pyruvate kinase M2 (PKM2) is a rate-limiting glycolytic enzyme that catalyzes the final step from phosphoenolpyruvate to pyruvate. Qi et al. reported that the expression and activity of PKM2 are upregulated in patients without DKD. Activation of PKM2 may protect against DKD by increasing glucose metabolic flux, inhibiting the production of toxic glucose metabolites, and inducing mitochondrial biogenesis to restore mitochondrial function [14]. Liu et al. demonstrated that PKM2 activation suppresses kidney fibrosis via inhibition of aberrant glycolysis associated with suppression of Hypoxia-inducible factor 1-alpha (HIF-1α) accumulation [15]. Gao et al. proved that PKM2 promotes pulmonary fibrosis by stabilizing TGF-β1 receptor I and enhancing TGF-β1 signaling [16]. Another rate-limiting glycolytic enzyme, phosphofructokinase (PFK), is a key enzyme in the glycolysis pathway, with three subtypes encoded by three different genes: liver type (PFKL), muscle type (PFKM), and platelet type (PFKP). The PFK catalyzes the irreversible conversion of fructose-6-phosphate (F6P) and ATP to fructose-1,6-bisphosphate (F-1,6-BP) and ADP during glycolysis, respectively. Transcription and translation of the PFKP gene can cause changes in glucose metabolism and affect the expression levels of multiple genes [17]. However, the role and molecular mechanism of PFKP in regulating PTECs energy metabolism remain to be largely elucidated.

In the present study, we investigated whether PFKP could mediate the progression of tubulointerstitial fibrosis by regulating metabolic reprogramming in PTECs and the mechanism by which TGF-β upregulates PFKP. By this mechanism, there may be a novel therapeutic target for CKD.

## Methods

### Animals and treatment

The animal procedures in this study were approved by the Animal Ethics Committee of the Second Clinical Medical College of Jinan University, Shenzhen People’s Hospital (Shenzhen, China),conforming to the Guide for the Care and Use of Laboratory Animals published by the NIH (NIH publication, eighth edition, updated 2011). The sample size for the animal studies was calculated based on a survey of data from published research or preliminary studies. Male C57BL/6 mice were purchased from the Animal Center of Nanjing University (Nanjing, Jiangsu, China) and housed in a temperature- and humidity-controlled environment with free access to food and drinking water. The mice were acclimatized for a 7-day period prior to the experiment. Mice were treated in a blinded fashion as the drugs used for treating animals were prepared by researchers who did not carry out the treatments. In addition, all animals were randomized before they received treatment. No mice were excluded from the statistical analysis. Studies were performed in accordance with the German Animal Welfare Act and reporting follows the ARRIVE guidelines [18].

In this study, healthy 8-week-old male mice were randomly allocated to four groups: sham, PFKP overexpression (PFKP OE)/AVV-*shPFKP*, UUO + AVV-Ctrl, and UUO + PFKP OE/AVV-*shPFKP*. For surgery, mice were subjected to left UUO or sham surgery, as previously described [19]. Briefly, mice were anesthetized with 2% pentobarbital sodium (4 ml/kg) by intraperitoneal injection. In the UUO group, the surgical procedure was performed on the left side of the abdomen. The procedure involved making two incisions, one through the skin and the other through the peritoneum, to expose the kidney. The left ureter was then ligated twice with surgical silk and severed between the two ligatures. The ligated kidney was repositioned and replenished with sterile saline. The incisions were sutured, and mice were individually housed. A sham operation was performed in a similar manner, but without ureteral ligation. Using CO2 inhalation, mice were euthanized at different time points post-surgery, and kidney tissues were harvested for pathological examination, immunohistochemistry, and other tests after perfusion with PBS.

To investigate whether isorhamnetin (ISO; Sigma-Aldrich, Hamburg, Germany) could alleviate the renal fibrosis in vivo, mice were randomly divided into five groups (*n* = 6 each) as follows: (i) the control group, C57BL/6J mice received sham surgery; (ii) the UUO group; C57BL/6J mice received UUO surgery treated with dimethyl sulfoxide only; (iii) the UUO group treated with 5 mg/kg ISO; (iv) the UUO group treated with 10 mg/kg ISO; (v) the UUO group treated with 30 mg/kg ISO. The dose of 10 mg/kg isorhamnetin treatment was chosen based on previous study where they studied the effect of isorhamnetin on GLUT4 levels in HFD-induced obesity mice model [20]. All treatments were given orally once per day. After 7 days of treatment, mice sacrificed using CO2 chamber, kidney were collected for ex vivo analysis.

### In situ injection of AAV9-*γ-GT*-*PFKP/shPFKP* into C57BL/6 J mice

In order to induce *PFKP* overexpression in mice, we utilized adeno-associated virus (AAV) serotype 9 (AAV9) vectors to introduce *PFKP* into the kidney with in situ injection. Specifically, we employed the proximal tubule-specific *paired box 8* promoter (AAV9-*γ-GT*) to deliver either AAV9-*γ-GT -PFKP* or AAV9-*γ-GT* -*Gfp* (GeneChem Company, Shanghai, China) in a volume of 200 μL saline (0.15 mol/L NaCl) at a concentration of 1 × 10^12^ vg/mL. Each group consisted of six C57BL/6 J mice. By utilizing this approach, we achieved our experimental objective while reducing the likelihood of plagiarism. In addition, we introduced the shRNA of *PFKP* (sh*PFKP*) into wild-type (WT) mouse kidneys using AAV9-*γ-GT*, and AAV9-*γ-GT* -*Gfp* transfection as control.

### Immunohistochemistry staining

Kidney specimens from mice were fixed using 4% paraformaldehyde and embedded in paraffin. Slices of 5 μm thickness were prepared and subjected to antigen retrieval and peroxidase removal. Subsequently, the slides were blocked using 5% goat serum and incubated overnight at −4 °C with antibodies against PFKP, α-SMA, phospho-S6 (p-S6), HIF-1α, and HK2. The sections were then incubated with secondary antibodies and subjected to DAB staining (ZSGB-BIO, Beijing, China; Cat. #PV-6000), followed by counterstaining with hematoxylin.

### Cell culture

The human proximal tubular epithelial cell line (HK2 cells) (Cat. #CRL-2190) were purchased from the American Type Culture Collection (Manassas, VA, USA). Cells were cultured in Minimum Essential Medium (Thermo Fisher Scientific, Shanghai, China; Cat. #10373017) containing 10% Fetal bovine serum (FBS), 100 U/mL penicillin, and 100 μg/mL streptomycin from Life Technologies (Carlsbad, CA, USA) at 37 °C in an incubator containing 5% CO_2_ and 95% air. Cells were regularly checked for mycoplasma in a standardized manner, by a qPCR test, performed under ISO17025 accreditation to ensure work was conducted in mycoplasma-negative cells.

### Histology

As reported in our previous study [19], mouse kidney tissue was embedded in paraffin to prepare sections for Masson trichrome staining (Solarbio Life Science, Beijing, China; Cat. # G1346) and Sirius Red staining with the Modified Sirius Red Stain Kit (Solarbio Life Science). The stained area was quantified using ImageJ software (NIH, http://rsbweb.nih.gov/ij/).

### Western blotting

Proteins were extracted from kidney tissues or HK2 cells by lysing snap-frozen tissues or cells on ice in RIPA buffer containing phosphatase and protease inhibitors (Beyotime Biotechnology, China; #P0013B, #P1005). Following centrifugation, the supernatant was collected and subjected to electrophoresis on 8–15% (depending on the target protein) sodium dodecyl sulfate-polyacrylamide gel electrophoresis gels and then transferred onto polyvinylidene difluoride (PVDF) membranes (Millipore, Bedford, MA, USA). The PVDF membranes were blocked with 5% milk and incubated overnight with primary antibodies (listed as antibodies and reagents). The following day, the PVDF membranes were incubated with the appropriate secondary antibodies and detected using chemiluminescence (Tanon, Shanghai, China). The immunoreactive bands were analyzed for density using ImageJ software.

### RNA extraction and qPCR or cell samples

TRIzol (Invitrogen) was used to extract total RNA from kidney tissues or cell samples according to the standard procedure. Using PrimeScript™ RT reagent Kit with gDNA Eraser (Takala, Beijing, China; Cat. #RR047A), RNA was reverse-transcribed to cDNA. qPCR was performed using TB Green® Premix Ex Taq™ II Mix (Takala, Cat. #RR820A) on a LightCycler 480 instrument (Roche). The primer sequences are listed in Supplemental Table S1. The data were analyzed using the ^ΔΔ^Ct-method, standardized to vinculin in kidney samples or GAPDH in cell samples.

### Antibodies and reagents

Antibodies against PFKP (cat. #ab119796), CTGF (cat. #ab209780), HIF-1α (cat. #ab179483), E-cadherin (cat. #ab231303) α-SMA (cat. # ab7817), hexokinase 2 (HEK2) (cat. #ab209847), SMAD3 (cat. #ab84177), p-SMAD3 (cat. #ab52903), SP1 (cat. #ab227383), PDK4 (cat. #ab214938), and β-actin were obtained from Abcam (Cambridge, UK). Antibodies against FN1 (cat. #63779S), phospho-PKM2 (p-PKM2) (Tyr105) (cat. #3827S), PKM2 (cat. #4053), and LDHA (cat. #2012S), anti-mouse or rabbit IgG antibody were purchased from Cell Signaling Biotechnology (MA, USA). COL1A1 (cat. #sc-59772), and COL3A1 (cat. #sc-271249) were obtained from Santa Cruz Biotechnology (Shanghai) Co. Ltd. Phosphor-LDHA (Tyr10) (cat. #PA5–105445) and the FBS was purchased from Invitrogen Life Technologies (Carlsbad, CA, USA).

### Immunofluorescence (IF)

Mouse kidney specimens were fixed in 4% paraformaldehyde and embedded in paraffin. From paraffin-embedded tissues, 5-μm-thick sections were cut and subjected to deparaffinization. Antigen retrieval was performed by incubating the sections in Target Retrieval Solution (Servicebio, Wuhan, China) buffer at 95 °C for 15 min. The sections were then incubated overnight at 4 °C with E-cadherin (1:100; Proteintech, Cat. #20874-1-AP). After washing, the sections were incubated with Alexa 488 fluorescent secondary antibody, and nuclei were counterstained with DAPI. The stained sections were visualized using confocal microscopy (Laica, Weztlar, Germany), and the area of staining was quantified using ImageJ software (NIH, http://rsbweb.nih.gov/ij/).

### Lactic acid determination in cells and primary mouse proximal tubule (PT) cells Isolation

Lactic acid determination in cells or mouse PT cell samples was carried out using an enzymatic lactate assay kit (Sigma-Aldrich) according to the standard procedure. Briefly, samples were centrifuged at 10,000 × *g* for 5 min at 4 °C. The supernatant was collected and deproteinized using perchloric acid. The resulting supernatant and standards were added to a 96-well microplate containing the lactate assay mix. The plate was then incubated at 37 °C for 30 min before measuring absorbance at 450 nm using a microplate reader. The lactate concentration was calculated accordingly.

To isolate primary mouse PT cells, we followed a previously described protocol [21]. The kidneys were harvested from the mice and mechanically dissociated using a GentleMACS cell dissociator (Miltenyl Biotec). We then isolated the cells using anti-Prominin-1 microbead-conjugated antibodies and autoMACS (Miltenyl Biotec).

### Extracellular acidification rate (EACR)

To assess the activity of cellular glycolysis, we measured the ECAR, which was calculated based on the pH change caused by the release of protons following lactate production in glycolysis. The ECAR values were obtained from real-time measurements using a Seahorse XF-24 extracellular flux analyzer and then normalized to the protein content of each sample. Statistical analysis was performed using GraphPad Prism software, and the results are presented as mean ± standard deviation (SD). The significance of differences between groups was determined using a two-tailed *t*-test, with a *p*-value of <0.05 considered as statistically significant.

### Chromatin immunoprecipitation (ChIP)

Using a commercial kit from Sigma-Aldrich (St. Louis, Missouri, USA), ChIP assays were performed following the manufacturer’s instructions with the primers: Forward Primer (5′→3′): ATGCTCCCGGCGTTCTAT; Reverse Primer (5′→3′): GCTGGAGGACTCTGGTTGG. Briefly, liver tissues were lysed in a lysis buffer and sonicated (15 s on and 90 s off, repeated eight times). After precipitation with Agarose A for 30 min, the fragmented DNA was pulled down with SMAD3 and SP1 antibodies or IgG and then subjected to amplification by qPCR.

### Dual-luciferase reporter assay

Human PFKP genes were amplified from cDNA extracted from HEK293T cells, a human embryonic kidney cell line, using PCR. The promoter fragments of the target genes were cloned using PCR and inserted into the pGL3 luciferase vector using the primers detailed in Table S2. All constructs were verified using DNA sequencing analysis. To conduct dual-luciferase reporter gene assays, HEK293T cells were transfected with the target gene promoter plasmids along with *PFKP* and Renilla luciferase. Firefly and Renilla luciferase activities were quantified using a dual-luciferase reporter gene system (Promega, Madison, WI, USA).

### Histopathological

Examination Tubular Injury Score Tubular injury score was estimated in renal tissue stained with hematoxylin and eosin, as described previously [22]. Briefly, renal tubular damage was graded on six levels on the basis of the loss of brush border, tubular dilation, cast formation, tubular necrosis, and neutrophil infiltration. Three high-power fields per mice (original magnification × 200) were chosen randomly; each field was scored from 0 to 5. All assessments were done by two investigators blinded to experimental conditions.

**Table:** 

0	Normal
1	mild injury, involvement of 0–10%
2	moderate injury, involvement of 11–25%
3	severe injury, involvement of 26–49%
4	high severe injury, involvement of 50–75%
5	extensive injury, involvement of 0.75%

### Data analysis

All data were obtained from at least five independent experiments. Each value is presented as the mean ± SD. All raw data were initially subjected to a normal distribution and analysis by a one-sample Kolmogorov-Smirnov nonparametric test using SPSS 22.0 software. For animal and cellular experiments, a two-tailed unpaired Student’s t-test was performed to compare two groups. One-way ANOVA followed by Bonferroni’s post-hoc test was used to compare more than two groups. The correlation coefficient was calculated using Spearman’s correlation test. To avoid bias, all statistical analyses were performed in a blinded manner. Statistical significance is indicated at *p < 0.05, **p < 0.01, and ***p < 0.001.

## Results

### In human and mice fibrotic kidneys, PFKP was significantly upregulated

**Fig. 1:**
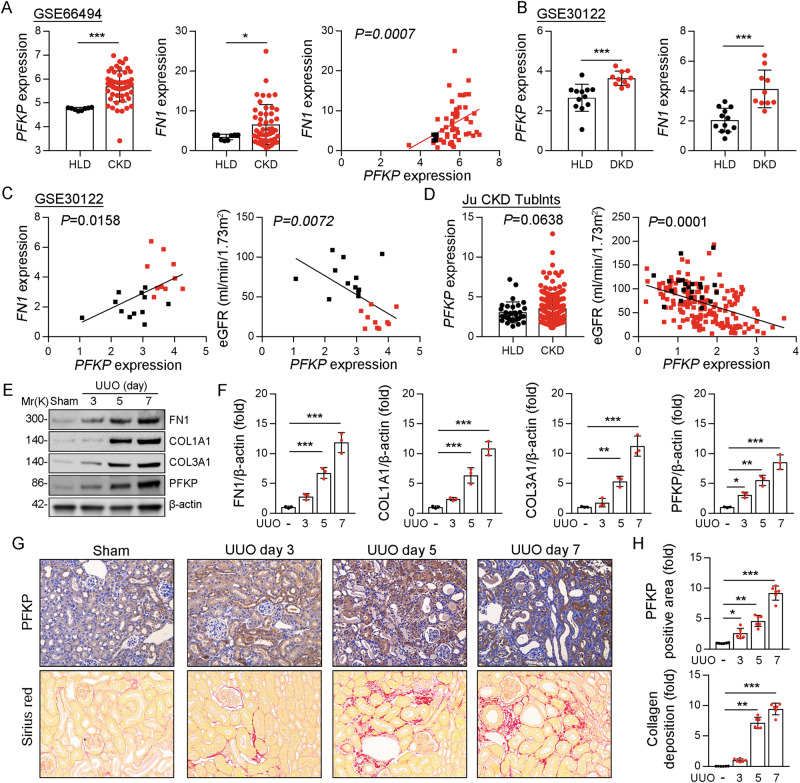
*PFKP* was significantly upregulated in human and mice fibrotic kidneys. **A** Reanalyses of microarray data on human renal biopsy specimens from patients with CKD (GSE66494) showed that the expression of *PFKP* and *FN1* in the kidney tissues, and the correlation between *PFKP* and *FN1*. **B** Reanalysis of microarray data obtained from renal biopsy specimens of patients with DKD (GSE30122) showed the expression levels of *PFKP* and *FN1* in renal tubules. **C** The correlation between *PFKP* and *FN1*, and between *PFKP* and eGFR (GSE30122). **D** The Ju CKD Tublnts database that covers many types of CKD showed the expression of *PFKP*, and the correlation between *PFKP* and eGFR in renal tubules. **E** Protein levels of PFKP and extracellular matrix (ECM) proteins (FN1, COL1A1, COL3A1) in the mouse renal tissues of UUO group compared with sham group. **F** The quantitative results of panel E are shown, *n* = 3 (β-actin was used as the loading control). **G**, **H** Immunohistochemistry of PFKP staining and Sirius red staining in the kidney tissues of UUO group compared with sham group (**G**). It was quantified in the kidney sections in 3 fields per mice at ×100 magnification (H), *n* = 6. Data are shown as means ± SD. *p < 0.05, **p < 0.01, ***p < 0.001.

Reanalysis of microarray data obtained from renal biopsy specimens of patients with CKD (GSE66494) showed that the expression levels of *PFKP* (*P* < 0.001) and *FN1* (a fibrogenic gene) were significantly increased in kidney samples from patients with CKD compared with those from healthy controls (Fig. 1A). There was a strong positive correlation between *PFKP* and *FN1* (Fig. 1A). In addition, reanalysis of microarray data obtained from renal biopsy specimens of patients with DKD (GSE30122) showed the same results; the expression levels of *PFKP* and *FN1* were significantly increased in renal tubules (Fig. 1B). There was a strong positive correlation between *PFKP* and *FN1*; however, there was a negative correlation between *PFKP* and eGFR (Fig. 1C). The Ju CKD Tublnt database [23] which covers many types of CKD also showed that *PFKP* and eGFR exhibit a negative correlation in the renal tubules (Fig. 1D). To investigate the potential involvement of PFKP in the pathogenesis of kidney fibrosis, we first determined its protein expression levels in fibrotic mouse kidney tissues induced by unilateral ureteral obstruction (UUO). As expected, immunoblotting showed significant upregulation of the protein expression levels of PFKP and extracellular matrix (ECM) proteins (FN1, COL1A1, and COL3A1) in the renal tissues of the UUO group compared with the sham group (Fig. 1E, F). Immunohistochemistry and Sirius red staining revealed a marked increase in the intensity of PFKP staining in the kidney tissues of the UUO group (Fig. 1G, H). Collectively, the observed upregulation of *PFKP* expression in fibrotic human or mouse kidneys and its positive correlation with fibrogenic ECM protein genes suggest a potential role of PFKP in the pathogenesis of kidney fibrosis.

### Overexpression of PFKP exacerbates renal fibrosis in the UUO mice model

**Fig. 2:**
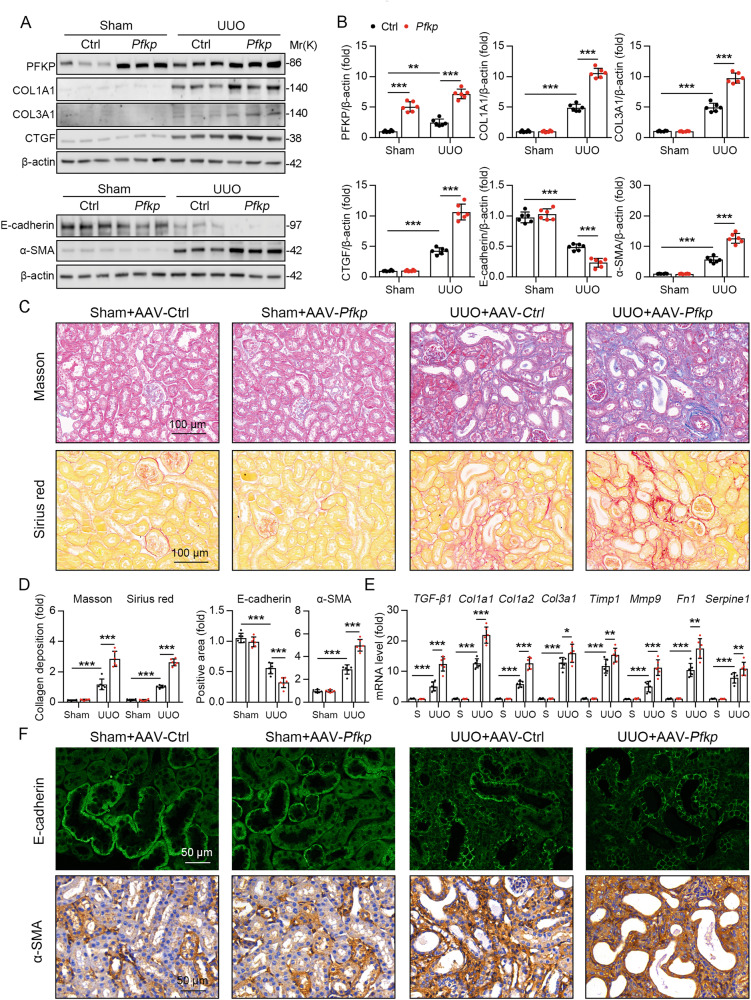
*PFKP* overexpression aggravated renal fibrosis in UUO mice. **A** Immunoblotting showed that the protein levels of *PFKP* and ECM proteins (COL1A1, COL3A1, and CTGF) in UUO mice group and sham group. Immunoblotting showed that the protein levels of the epithelial marker E-cadherin and the mesenchymal marker α-SMA, *n* = 6. **B** The quantitative results of panel A are shown, *n* = 6 (β-actin was used as the loading control). **C** Masson staining and Sirius red staining showed that severity of renal fibrosis was more significant in UUO mice group and sham group. **D** (Left panel) For masson’s trichrome staining and Sirius red staining was quantified in the kidney sections in 3 fields per mice at ×100 magnification, *n* = 6. (Right panel) For immunofluorescence staining detection for E-cadherin and immunohistochemistry staining detection for α-SMA was quantified in the kidney sections in 3 fields per mice at ×100 magnification, *n* = 6. **E** The mRNA expression levels of *Tgf-β1, Col1α1, Col1α2, Col3α1, Tmip 1, Mmp9, Fn1*, and *Serpine1* in the renal tissues of mice in UUO group compared with sham group, *n* = 6. **F** Immunofluorescence staining detection for E-cadherin and immunohistochemistry staining detection for α-SMA in the kidney of mice, 3 fields per mice at ×200 magnification. Data are shown as means ± SD; *p < 0.05, **p < 0.01, ***p < 0.001.

We introduced PFKP into WT mouse kidneys using AAV9-*γ-GT*, and AAV9-*γ-GT* -*Gfp* transfection as a control (Fig. 2A). After 2 weeks of AAV transfection, WT mice were subjected to UUO surgery. Immunoblotting showed that overexpression of *Pfkp* promoted the expression of ECM proteins (COL1A1, COL3A1, and CTGF) in UUO mice (Fig. 2A, B). Masson staining and Sirius red staining showed that, compared to the sham surgery group, renal fibrosis was more significant in the UUO mice group, and the degree of fibrosis was more severe after overexpression of *Pfkp* (Fig. 2C, D).

Furthermore, the mRNA expression levels of *Tgf-β1, Col1α1, Col1α2, Col3α1, Tmip1, Mmp9, Fn1*, and *Serpine1* were significantly elevated in the renal tissues of mice in the UUO group compared with those in the sham group, and further increased after overexpression of *Pfkp* (Fig. 2E). In addition, immunoblotting and immunohistochemistry revealed that the epithelial marker E-cadherin was suppressed and the mesenchymal marker α-SMA was upregulated after overexpression of *PFKP*, suggesting that PFKP induced epithelial-mesenchymal transition (EMT) in PTECs (Fig. 2A, D, F). According to the H&E staining, we found that PFKP overexpression aggravated UUO-induced tubular injury (Fig. S1A). In agreement with this, the tubular damage markers neutrophil gelatinase-associated lipocalin (NGAL) and kidney damage molecule-1 (KIM-1) was upregulated in UUO mice and further increase after PFKP overexpression (Fig. S1A). Collectively, these results suggest that overexpression of *Pfkp* exacerbates renal fibrosis in the UUO mouse model.

### Knocking down PFKP attenuate renal fibrosis in UUO mice model

**Fig. 3:**
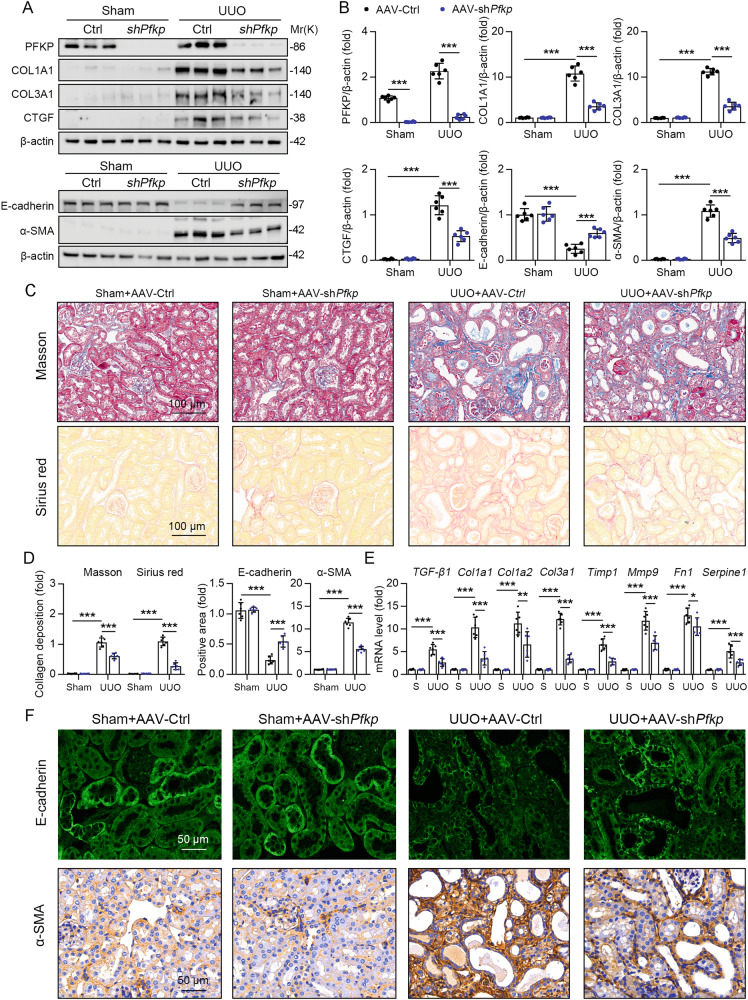
Knocking down *PFKP* attenuate renal fibrosis in UUO mice model. **A** Immunoblotting showed that the protein levels of *PFKP* and ECM proteins (COL1A1, COL3A1, and CTGF) in UUO mice group and sham group. Immunoblotting showed that the protein levels of the epithelial marker E-cadherin and the mesenchymal marker α-SMA. **B** The quantitative results of **A** are shown, *n* = 6 (β-actin was used as the loading control). **C** Masson staining and Sirius red staining showed that severity of renal fibrosis was more significant in UUO mice group and sham group. **D** (Left panel) For masson’s trichrome staining and Sirius red staining was quantified in the kidney sections in 3 fields per mice at ×100 magnification, *n* = 6. (Right panel) For immunofluorescence staining detection for E-cadherin and immunohistochemistry staining detection for α-SMA was quantified in the kidney sections in 3 fields per mice at ×100 magnification, *n* = 6. **E**. The mRNA expression levels of *Tgf-β1, Col1α1, Col1α2, Col3α1, Tmip 1, Mmp9, Fn1*, and *Serpine1* in the renal tissues of mice in UUO group compared with sham group, *n* = 6. **F** Immunofluorescence staining detection for E-cadherin and immunohistochemistry staining detection for α-SMA in the kidney of mice, 3 fields per mice at ×200 magnification. Data are shown as means ± SD; *p < 0.05, **p < 0.01, ***p < 0.001.

Next, we introduced sh*Pfkp* into WT mouse kidneys using AAV9-*γ-GT*, and AAV9-*γ-GT*-*Gfp* transfection as control (Fig. 3A). After 2 weeks of AAV transfection, mice were subjected to UUO surgery. Immunoblotting showed that knockdown of *Pfkp* suppressed the expression of ECM proteins (COL1A1, COL3A1, and CTGF) in UUO mice (Fig. 3A, B). Masson staining and Sirius red staining showed that compared to the sham surgery group, renal fibrosis was more significant in the UUO mice group, and knocking down *Pfkp* significantly reduced the degree of renal fibrosis (Fig. 3C, D). Furthermore, the mRNA expression levels of *Tgf-β1, Col1α1, Col1α2, Col3α1, Tmip 1, Mmp9, Fn1*, and *Serpine1* were significantly increased in the renal tissues of mice in the UUO group compared with those in the sham group, but reduced after knocking down *Pfkp* (Fig. 3E). In addition, immunoblotting and immunohistochemistry revealed that the epithelial marker E-cadherin was upregulated and the mesenchymal marker α-SMA was suppressed after knocking down *Pfkp* (Fig. 3A, D, F). According to the H&E staining, we found that PFKP knockdown alleviated UUO-induced tubular injury (Fig. S1B). Furthermore, the mRNA level of *Ngal* and *Kim-1* was increased in UUO mice, but decreased after PFKP knockdown (Fig. S1B). Collectively, these results suggest that knockdown of *Pfkp* attenuates renal fibrosis in the UUO mouse model.

### PFKP regulates renal glycolysis

**Fig. 4:**
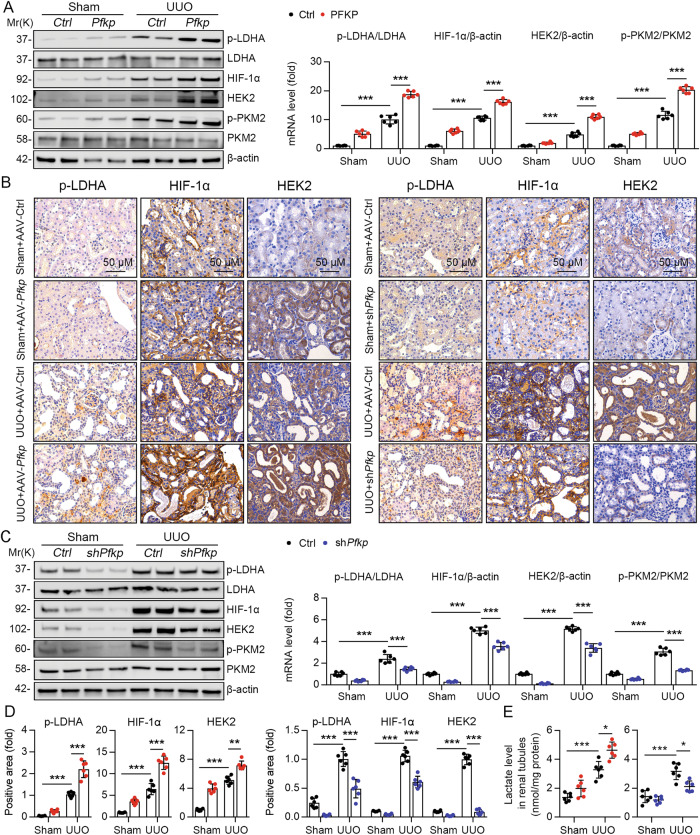
PFKP regulates renal glycolysis. **A**, **C** Immunoblotting revealed the expression of glycolysis-related genes such as phospho-LDHA, HIF-1α, HEK2, and phospho-PKM2 in the renal tissues of mice in UUO group compared with sham group. The quantitative results of western blot are shown in the right panel, *n* = 6 (β-actin was used as the loading control). **B** Immunohistochemistry showed that phospho-LDHA, HIF-1α, and HEK2 in the renal tissues of mice in UUO group compared with sham group. **D** For immunohistochemistry staining detection for p-LDHA, HIF-1α, and HEK2 was quantified in the kidney sections in 3 fields per mice at ×100 magnification, *n* = 6. **E** Lactate concentrations in renal tubule cells from UUO group and sham group, *n* = 6. Data are shown as means ± SD; *p < 0.05, **p < 0.01, ***p < 0.001.

Energy reprogramming to glycolysis is closely associated with CKD development. PFKP is the rate-limiting enzyme in glycolysis. Therefore, we investigated the role of PFKP in the regulation of renal glycolysis in vivo. We analyzed the protein expression of the key enzymes of glycolysis in the UUO and sham groups. Immunoblotting and immunohistochemistry analysis revealed that the expression of glycolysis-related genes, such as phospho-LDHA, HIF-1α, HEK2, and phospho-PKM2, was significantly elevated in the renal tissues of mice in the UUO group compared with the sham group, and further increased after overexpression of *Pfkp* (Fig. 4A, B). Conversely, knockdown of *Pfkp* showed the opposite results; the expression of glycolysis-related genes was significantly decreased (Fig. 4B, C). Furthermore, lactate concentrations were obviously increased in PTECs from the UUO group and further elevated after overexpression of *Pfkp* (Fig. 4E). However, lactate concentrations in PTECs of UUO mice were significantly decreased after knockdown of *Pfkp* (Fig. 4E).

### PFKP plays an important role during TGF-β-induced glycolysis in PTECs

**Fig. 5:**
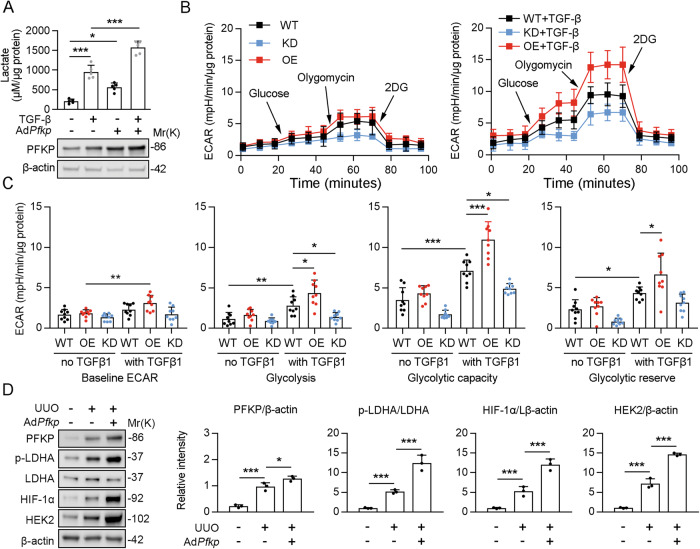
PFKP plays an important role during TGF-β1-induced glycolysis in renal TECs. **A** Lactate production in PFKP overexpression or naive HK2 cells in the absence or presence of TGF-β1, *n* = 5. Immunoblotting showed that the protein levels of PFKP. **B**, **C** WT, PFKP overexpression (OE), and PFKP knockdown (KD) cells were seeded in Seahorse XF-24 cell culture microplates. The cells were rendered quiescent in 0.5% FBS DMEM overnight and then treated with or without 10 ng/ml TGF-β1 for 24 h. All the cells were incubated in the glycolysis stress test medium without glucose and pyruvate, followed by sequential treatments with glucose (10 mM), oligomycin (5 μg/ml), and 2-deoxyglucose (2-DG; 50 mM). Real-time extracellular acidification rate (ECAR) was recorded as the baseline (before glucose), the rate of glycolysis (after glucose), glycolytic capacity (after oligomycin), and glycolytic reserve (after 2-DG); *n* = 9. **D** The PTECs isolated from the mice treated as indicated in the Fig. 2. Immunoblotting showed that the protein levels of PFKP, p-LDHA, LDHA, HIF-1α, HEK2, and β-actin, and the quantitative results of western blot are shown in the right panel, *n* = 3 (β-actin was used as the loading control). Data are shown as means ± SD; *p < 0.05, **p < 0.01, ***p < 0.001.

It is well accepted that the TGF-β signaling pathway plays a crucial role in fibrogenesis, especially in renal fibrosis in CKD [24, 25]. Srivastava et al. confirmed that TGF-β1 induces glycolysis in human PTECs [26]. We further analyzed whether PFKP was involved in the regulation of glycolysis activity by TGF-β in HK2 cells (human PTECs line). We found that PFKP overexpression significantly increased lactate production with or without TGF-β1 stimulation (Fig. 5A). We then performed a glycolysis stress test using the Seahorse X24 extracellular flux analyzer. All three types of cells (WT; *PFKP* overexpression, OE; and *PFKP* knockdown, KD), treated with or without TGF-β1, were incubated in the glycolysis stress test medium (no glucose and pyruvate). Next, the cells received serial exposures to glucose, oligomycin, and 2-deoxyglucose (2-DG; a glucose analog that inhibits glycolysis through competitive binding to HEK2, the first enzyme in the glycolytic pathway; Fig. 5B). The baseline ECAR, the rate of glycolysis, glycolytic capacity, and glycolytic reserve were determined. Without TGF-β1 treatment, *PFKP* overexpression did not change the baseline ECAR, the rate of glycolysis, glycolytic capacity, or glycolytic reserve. Interestingly, *PFKP* knockdown significantly decreased glycolytic capacity and reserve compared with WT cells (Fig. 5B, C). In WT cells, TGF-β1 significantly increased glycolysis, glycolytic capacity, and glycolytic reserve (Fig. 5B, C). This effect of TGF-β1 on ECAR was significantly enhanced by *PFKP* overexpression and reduced by *PFKP* knockdown (Fig. 5B, C). Of note, unlike the lactate assay, PFKP OE cells did not increase glycolysis without TGF-β1 stimulation (Fig. 5B, C). This was likely due to the fact that the base medium used in Seahorse experiments did not have glucose and pyruvate. Next, we wonder verified that the expression of glycolysis-related genes in PTECs after kidney injury. Our results showed that the the expression of glycolysis-related genes, such as phospho-LDHA, HIF-1α, and HEK2, was significantly elevated in the PTECs isolated from UUO mice, and further increased after overexpression of *Pfkp* (Fig. 5D). Collectively, PFKP was involved in TGF-β1-mediated upregulation of glycolysis capacity in HK2 cells.

### TGF-β1 upregulates the transcription level of PFKP by recruiting the SMAD3-SP1 complex to the PFKP promoter

**Fig. 6:**
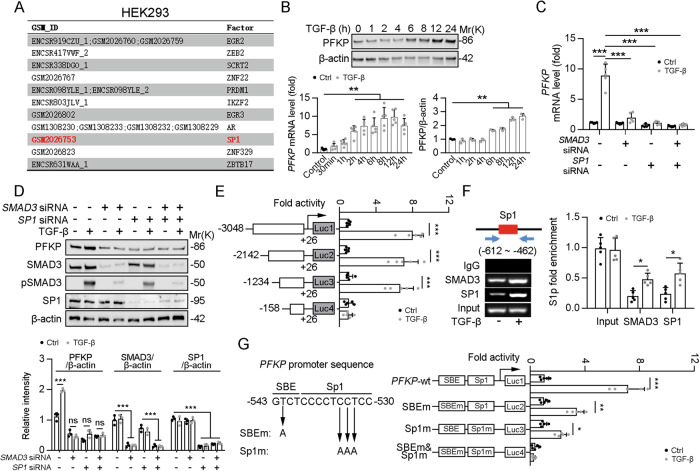
TGF-β stimulation upregulated PFKP expression. **A** Reanalyzed Chip-seq database from Cistrome Data Browser. **B** Immunoblotting showed that the protein levels of *PFKP* in the HK2 cells treated with TGF-β1 (2 ng/mL) as indicated in the figure (upper panel). The quantitative results of western blot are shown in the right of bottom panel, *n* = 3 (β-actin was used as the loading control). The mRNA expression levels of *PFKP* in the HK2 cells treated with TGF-β1 (2 ng/mL) as indicated in the figure (the left of bottom panel). **C** HK2 cells treated with SMAD3 siRNA and SP1 siRNA alone or combined under TGF-β1 stimulation, and the mRNA level of *PFKP* were detected by qPCR, *n* = 5. **D** HK2 cells treated with SMAD3 siRNA, SP1 siRNA, and TGF-β1 as indicated in the figure. Immunoblotting showed that the protein levels of SMAD3, phosphorylation of SMAD3, SP1, and PFKP in the HK2 cells. The quantitative results of western blot are shown in the bottom panel, *n* = 3 (β-actin was used as the loading control). **E** A series of truncated PFKP promoters fused to the luciferase reporter gene was cotransfected into HEK293T cells together with a Renilla plasmid, followed by TGF-β stimulation or not, *n* = 3. **F** HK2 cells were incubated with TGF-β for 24 h. DNA fragments containing the flanking regions of Sp1 on the *PFKP* promoter were immunoprecipitated with anti-SMAD3 or anti-SP1 and then PCR amplified, as shown in the bottom panel, *n* = 5. **G** Nucleotide sequence of the −543 to −530 segment of the *PFKP* promoter. Predicted SP1 binding sites (Sp1), SBE, and their mutations (SBEm and Sp1m) are indicated (left panel). Sp1 sites and SBEs were mutated individually or in combination in the *PFKP* promoters fused to the luciferase reporter gene and cotransfected into HEK293T cells together with a Renilla plasmid, followed by TGF-β stimulation (*n* = 3).

The Cistrome DB Toolkit facilitates searches for factor binding, histone modifications, and chromatin accessibility in any given genomic interval shorter than 2 Mb [27]. Hence, we employed the Cistrome DB Toolkit to search for transcript factor binding to the *PFKP* promoter, and SP1 was predicted to directly bind to the *PFKP* promoter (Fig. 6A). As is well known, TGF-β signaling regulates downstream gene expression via formation of complexes in the nucleus between SMADs and DNA-binding cofactors, such as SP1, or with transcriptional coactivators or corepressors [28]. Indeed, TGF-β treatment increased the mRNA and protein levels of PFKP in HK2 cells (Fig. 6B). Furthermore, TGF-β-induced upregulation of PFKP was significantly inhibited by siRNA SMAD3 and siRNA SP1 individually or in combination (Fig. 6C, D). We cloned a series of *PFKP* promoter deletion mutants into the luciferase reporter system (Luc1–Luc4; Fig. 6E) and found that TGF-β stimulation enhanced PFKP activity for the Luc1–3 construct, indicating that the minimal SMAD3-SP1 complex binding site within the PFKP promoter was between −1234 and −158 bp. We further performed ChIP assays and found that SMAD3 and SP1 were recruited to the −612 to −462 bp region of the *PFKP* promoter (Fig. 6F). Next, we defined the contributions of the SMAD3 and SP1 binding elements to basal and TGF-β-induced transcription from the PFKP promoter. Mutations were introduced to abolish the binding of SMAD3 or SP1 (Fig. 6G). Our results showed that mutations of individual SMAD binding element (SBE) also inhibited TGF-β-induced transcription levels, but there was no significant effect on the basal transcription levels. Furthermore, mutations of SP1 decreased the TGF-β-induced transcription, whereas mutations in both sites decreased the basal transcription levels and abolished TGF-β inducibility (Fig. 6G). Collectively, TGF-β1 enhances the transcription level of *PFKP* by recruiting the SMAD3-SP1 complex to the *PFKP* promoter.

### Isorhamnetin inhibits renal PTECs glycolysis by suppressing TGF-β-induced PFKP expression

**Fig. 7:**
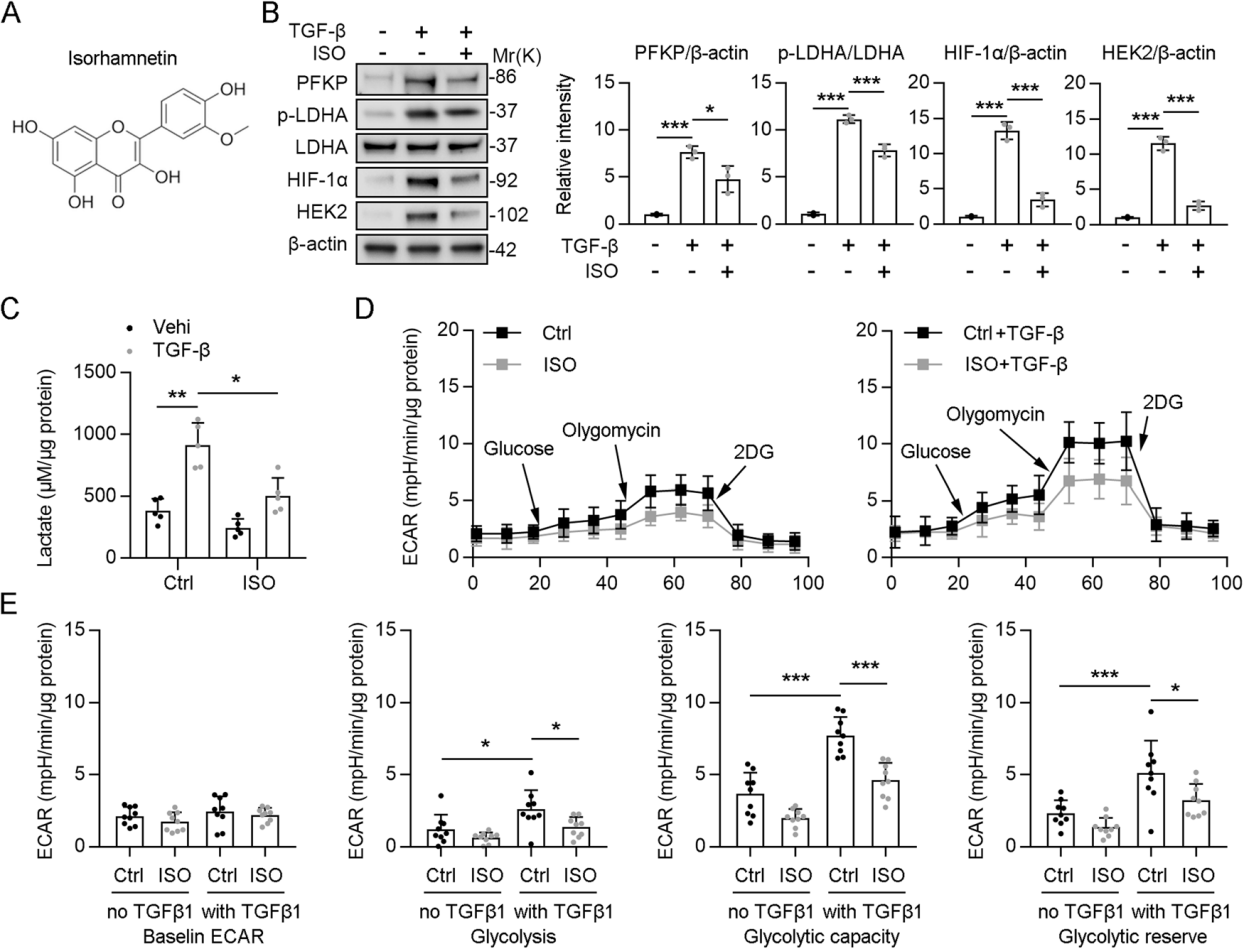
Isorhamnetin inhibits renal TECs glycolysis by suppressing TGF-β-induced *PFKP* expression. **A** Chemical structure of isorhamnetin (ISO). **B** The protein level of PFKP, p-LDHA, LDHA, HIF-1α, HEK2, and β-actin after ISO treatment in the absence or presence of TGF-β1, and the quantitative results of western blot are shown in the right panel, *n* = 3 (β-actin was used as the loading control). **C** Lactate production after ISO treatment in the absence or presence of TGF-β1 in HK2 cells, *n* = 5. **D**, **E** HK2 cells were treated with ISO or vehicle, and were seeded in Seahorse XF-24 cell culture microplates. The cells were rendered quiescent in 0.5% FBS DMEM overnight and then treated with or without 10 ng/ml TGF-β1 for 24 h. All the cells were incubated in the glycolysis stress test medium without glucose and pyruvate, followed by sequential treatments with glucose (10 mM), oligomycin (5 μg/ml), and 2-deoxyglucose (2-DG; 50 mM). Real-time extracellular acidification rate (ECAR) was recorded as the baseline (before glucose), the rate of glycolysis (after glucose), glycolytic capacity (after oligomycin), and glycolytic reserve (after 2-DG); *n* = 9. Data are shown as means ± SD; *p < 0.05, **p < 0.01, ***p < 0.001.

In our previous study, we found that the total flavone of Astragalus membranaceus (TFA) can attenuate atherosclerosis via dual suppression of miR-33 and the NF-κB pathway, and partially through inhibition of scavenger receptors in macrophages. The main ingredients of TFA include calycosin, kaempferol, Isoliquiritigenin, Isorhamnetin (ISO; Fig. 7A), formononetin, methylnissolin, isomucronulatol, and quercetin [29]. Among them, we found that only ISO suppressed the TGF-β-mediated upregulation of *PFKP* as well as glycolysis-related genes, such as phosphor-LDHA, HIF-1α, and HEK2 (Fig. 7B). Predominantly, ISO (C16H12O7) is a flavone found in sea buckthorn and ginkgo fruits. It exhibits various biological activities, including anti-inflammatory, anticancer, antioxidant, and antibacterial activities [30]. In addition, ISO exerts protective effects against cardiovascular and neurodegenerative diseases [31, 32]. It also has pharmacodynamic effects against hyperuricemia, acute kidney injury, and pulmonary fibrosis [33–35]. The pharmacological effects of ISO are related to its regulation of NF-κB, PI3K/AKT, MAPK, and other signaling pathways and their downstream factors [36]. We then tested the lactate concentration of HK2 cells. Increased lactate concentration was evident in HK2 cells incubated with TGF-β1, and lactate concentration was suppressed by ISO (Fig. 7C). Furthermore, we used ECAR to assess the activity of cellular glycolysis. The TGF-β1 treatment led to an immediate and significant increase in ECAR, thereby reflecting an enhancement of the acute glycolytic response in HK2 cells. However, EACR was significantly reduced after ISO treatment, reflecting an impaired glycolytic response in HK2 cells (Fig. 7D, E). According to the H&E staining, we found that ISO treatment alleviated UUO-induced tubular injury (Fig. S1C). In addition, the mRNA level of *Ngal* and *Kim-1* was increased in UUO mice, but reduced by ISO treatment (Fig. S1C). Collectively, these results suggest that ISO inhibits glycolysis by suppressing TGF-β-induced PFKP expression, at least in part, in HK2 cells.

### In UUO mice, ISO attenuates renal fibrosis and glycolysis

**Fig. 8:**
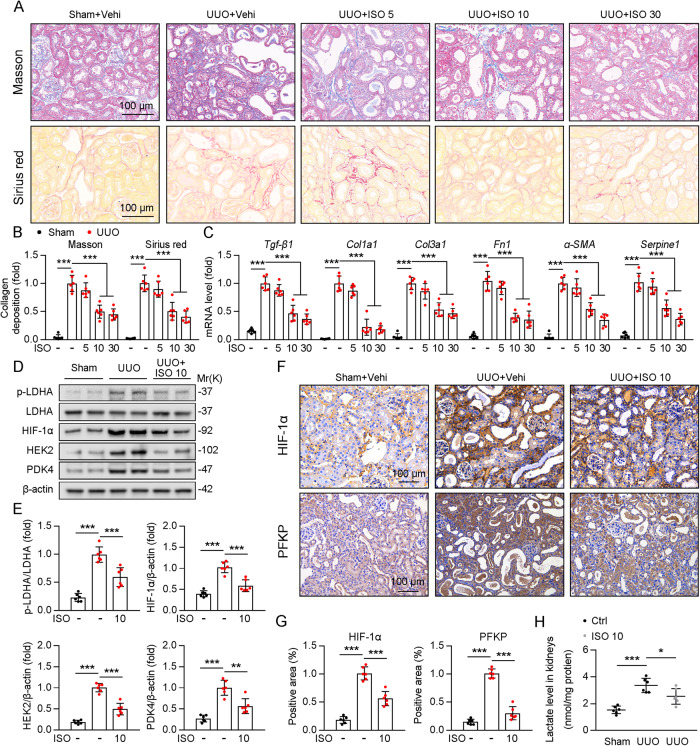
ISO attenuates renal fibrosis and glycolysis in UUO mice. **A** Masson staining and Sirius red staining showed that severity of renal fibrosis in UUO mice group and sham group with or without ISO treatment, 3 fields per mice at 200 × magnification, *n* = 6. **B** For masson’s trichrome staining and Sirius red staining was quantified in the kidney sections in 3 fields per mice at ×100 magnification, *n* = 6. **C** The mRNA expression levels of *Tgf-β1, Col1α1, Col3α1, Fn1, α-SMA* and *Serpine1* in the renal tissues of mice in UUO group compared with sham group with or without ISO treatment, *n* = 6. **D** Immunoblotting revealed the expression of glycolysis-related genes such as phospho-LDHA, HIF-1α, HEK2, and PDK4 in the renal tissues of mice in UUO group compared with sham group with or without ISO treatment. **E** The quantitative results of western blot of panel D are shown, *n* = 3 (β-actin was used as the loading control). **F** Immunohistochemistry staining detection for HIF-1α and PFKP with or without ISO treatment, 3 fields per mice at ×200 magnification, *n* = 6. **G** For immunohistochemistry staining detection for HIF-1α, and PFKP was quantified in the kidney sections in 3 fields per mice at ×100 magnification, *n* = 6. **H** Lactate production in TECs from UUO group and sham group with or without ISO treatment, *n* = 6. Data are shown as means ± SD; *p < 0.05, **p < 0.01, ***p < 0.001.

Next, we determined whether ISO intervention inhibited renal fibrosis and elevated glycolysis in the kidneys of UUO mice. Masson staining and Sirius red staining showed that renal fibrosis was more significant in UUO mice than in sham surgery mice, and the degree of renal fibrosis was significantly reduced after treatment with ISO at dose of 10 and 30 mg/kg (Fig. 8A, B). In line with this, the mRNA levels of *Tgf-β1, Col1a1, Col3a1, Fn1, α-SMA*, and *Serpine1* increased in UUO mice compared to sham mice, and this upregulation was inhibited after ISO intervention (10 and 30 mg/kg) (Fig. 8C). We found that 10 and 30 mg/kg doses of ISO had comparable inhibitory effects on renal fibrosis, so subsequent validation trials were conducted primarily in the 10-dose group. Immunoblotting analysis revealed that the expression of glycolysis-related genes such as phospho-LDHA, HIF-1α, HEK2, and PDK4 was significantly elevated in the renal tissues of mice in the UUO group compared with the sham group and further suppressed after treatment with ISO (Fig. 8D, E)*.* Notably, immunohistochemistry showed that renal HIF-1α and PFKP deposits were significantly increased in the UUO group compared with those in the control group, which was improved after treatment with ISO (Fig. 8F, G). Furthermore, consistent with the in vitro findings, the lactate concentration was also suppressed by the ISO intervention (Fig. 8H). Collectively, these data suggest that ISO attenuates renal fibrosis and glycolysis in vivo.

### FPKP promoted UUO-induced renal fibrosis dependent glycolysis

We overexpressed PFKP in UUO mouse kidneys and treated them with 2-DG to observe the effect of PFKP overexpression on renal fibrosis under conditions of glycolysis inhibition. Our results showed that the significant fibrosis induced by PFKP overexpression in mouse kidneys was greatly inhibited under 2-DG treatment (Fig. S2A, B). Notably, based on the expression of fibrosis-related genes, Masson and Sirius Red staining, we found that the combined treatment of PFKP + 2-DG did not significantly induce renal fibrosis compared to UUO mice treated with AAV-ctrl and vehicle (Fig. S2C, D). In line with this, FPKP overexpression induced the mRNA levels of *Tgf-β1, Timp1, Mmp9, Col1a1, Col1a2, Col3a1, and Fn1* was dramatically repressed after 2-DG treatment (Fig. S2E). Next, we found that 2-DG restrained FPKP ovexrepssion-induced tubular injury (assessed by H&E staining, and the mRNA level of *Nagl* and *Kim-1*) in UUO mice (Fig S1D). These results suggest to some extent that PFKP in the kidneys primarily influences renal fibrosis through its impact on glycolysis.

## Discussion

To maintain their normal functions, PTECs have high energy demands. The energy requirements of these cells are primarily satisfied by ATP generated via oxidative phosphorylation (OXPHOS), and FAO contributes about 70% of the total supply. However, in the case of diabetes, cell metabolism changes from FAO to glycolysis or its side branches in PETCs [11]. Indeed, treatment of mice with the glycolysis inhibitor 2-DG ameliorated PTECs proliferation, cystogenesis, and kidney fibrosis [37]. In addition, metformin can activate AMPK, promote acetyl-CoA carboxylase phosphorylation, upregulate FAO, and improve renal fibrosis [38]. Cai et al. found that dapagliflozin (sodium-glucose cotransporter 2 inhibitor; SGLT2i) suppressed the HIF-1α-mediated metabolic switch from lipid oxidation to glycolysis in kidney tubule cells of diabetic mice and significantly improved diabetes-induced tubulointerstitial damage, such as macrophage infiltration and fibrosis [9]. Li et al. also confirmed that empagliflozin (SGLT2i) normalized the suppressed SIRT3 levels, aberrant glycolysis, and inhibition of EMT in PTs [39]. Moreover, fenofibrate was found to be the dominant medication effector of PPARα-regulated FAO induction in the PT, and its gene targets and related urinary metabolites were moderately to strongly correlated with improvements in glomerular and proximal tubular structural parameters [40]. In the current study, we observed that PFKP knockdown alleviated glycolysis and interstitial fibrosis in the kidney after UUO surgery. Our data further support the notion of a metabolic shift to glycolysis in PTECs during the progression of renal fibrosis.

In the glomerulus, podocytes exhibit a strong preference for anaerobic glycolysis and are almost independent of mitochondrial OXPHOS to generate energy [41]. Indeed, it has been reported that the expression of PFKP is increased in the glomeruli of patients with DKD, and PFKP activation ameliorates podocyte cytoskeletal remodeling in podocytes through regulation of F-1,6-BP levels and inhibition of the Ras homology A/Rho-associated kinase 1 (RhoA/ROCK1) pathway in vitro [42]. Exogenous F-1,6-BP addition reduced foot process fusion and renal damage in *db/db* mice. These findings provide evidence that PFKP may be a potential target for podocyte injury in DKD [42]. In contrast to podocytes, PTECs rely almost entirely on mitochondrial FAO to generate energy [41]. During CKD, PTECs undergo metabolic reprogramming to activate glycolysis and its branches, and its metabolites promote the production of ROS [43]. Moreover, enhanced glycolysis in PTECs induces EMT and exacerbates renal fibrosis [44]. As previously mentioned, inhibiting glycolysis in PTECs can improve interstitial fibrosis. In line with this, in the present study, we found that overexpression of *PFKP* in PTECs exacerbates TGF-β-induced glycolysis and renal fibrosis, while knocking down *PFKP* attenuates PTECs glycolysis and renal fibrosis in vitro and in vivo. Since healthy PTECs use fatty acids as the main energy substrate, whereas podocytes exhibit a strong preference for anaerobic glycolysis, PFKP plays different roles in glomerular and tubular cells.

In recent years, plant extracts and their bioactive phytochemistry have attracted attention as new therapeutic and preventive alternatives for T2DM and its associated complications. Previously, ISO has long been studied for its potential antidiabetic effects [45, 46]. Emerging evidence suggests that ISO can improve diabetes-related disorders by decreasing glucose levels, ameliorating oxidative status, alleviating inflammation, and modulating lipid metabolism and adipocyte differentiation by regulating the involved signaling pathways [47]. However, the protective mechanism of ISO on the progression of renal fibrosis is still unclear. In the present study, we found that ISO could inhibit the expression of PFKP and decrease glycolysis and renal fibrosis in UUO mice (Figs. 7, 8), suggesting that ISO may be a new treatment strategy for renal fibrosis.

In summary, we found that PFKP played a critical role in the metabolic switch toward glycolysis in PTECs during the development of fibrosis. Deficiency of PFKP repressed, while PFKP overexpression promoted, the dilated tubules, the area of interstitial fibrosis, and glycolysis in the kidney of mice following injury. Mechanistically, TGF-β1 increased the transcription level of *PFKP* by recruiting the SMAD3-SP1 complex to the *PFKP* promoter in HK2 cells. Furthermore, inhibition of glycolysis and PFKP expression by ISO ameliorated renal fibrosis. These results provide a basis for further exploration of the therapeutic potential of targeting PFKP in CKD.

### Limitation

First, we did not validate the role of PFKP in other renal fibrosis models, such as folic acid-induced AKI models and unilateral renal ischemia-reperfusion models. Second, we did not adopt more AKI-CKD models, such as 5/6 nephrectomy and unilateral ischemia-reperfusion injury (28 d) models. Lastly, we did not construct and validate mice with tubule-specific knockout of PFKP.

### Supplementary information

Supplemental Material

Original Data

Reproducibility checklist

## Data Availability

The data that support the findings of this study are available from the corresponding author upon reasonable request.
