# Supplementary material for: Inhibition of PFKP in renal tubular epithelial cell restrains TGF-β induced glycolysis and renal fibrosis
Source: Cell Death Dis. 2023 Dec 12;14(12):816. doi: 10.1038/s41419-023-06347-1 (PMC10716164; doi:10.1038/s41419-023-06347-1)
Supplement: Supplementary file 1 — Supplemental Material [file 41419_2023_6347_MOESM1_ESM.docx]

**Electronic Supplementary Figures and Tables**

***Supplementary figure***

***
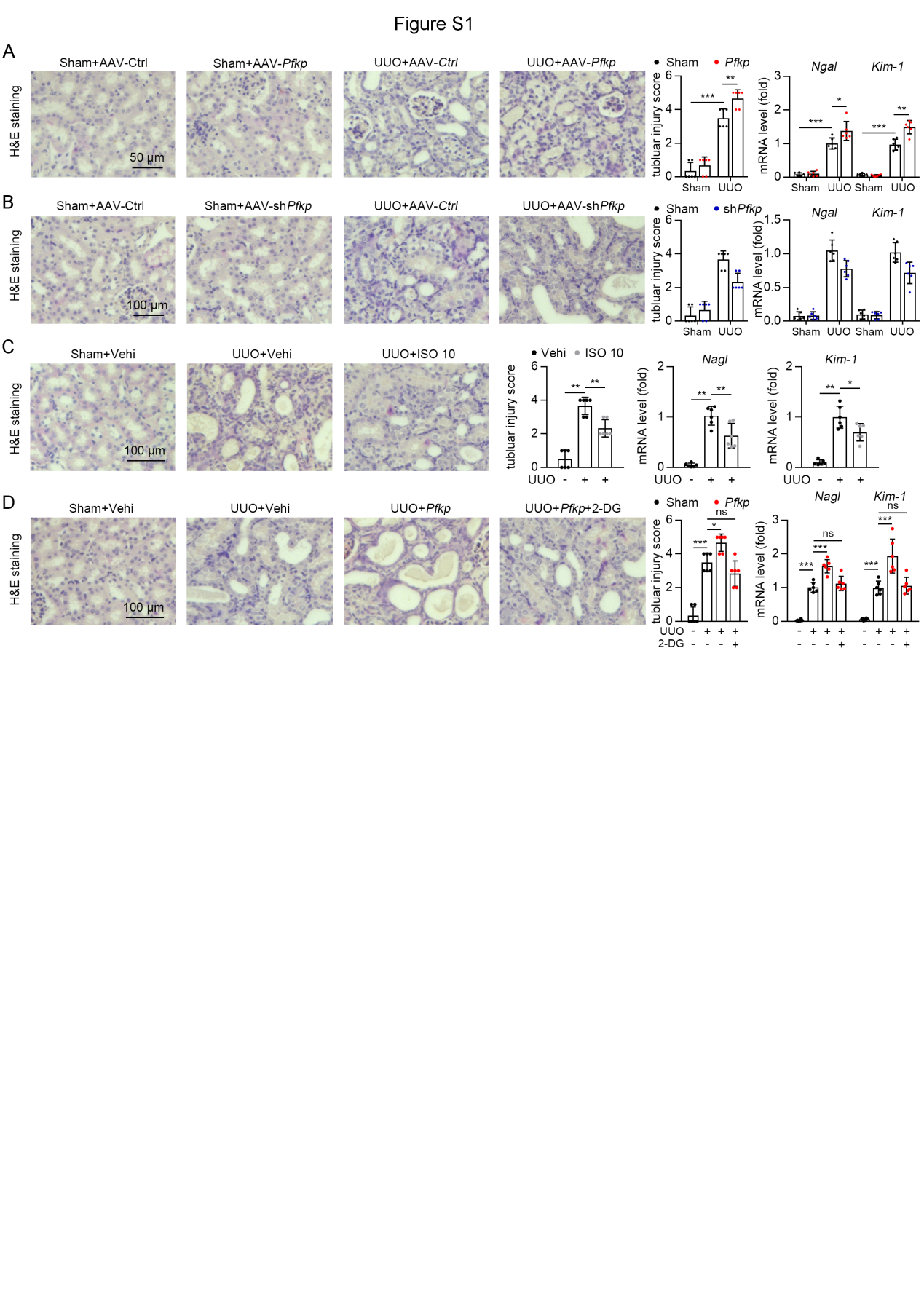
***

**Figure S1.** **PFKP aggravated UUO-induced tubular injury. A - D.** Representative hematoxylin and eosin (H&E)–stained renal sections from mice were shown in the left panel treated as indicated in the figure. Middle panel: mean tubular injury scores
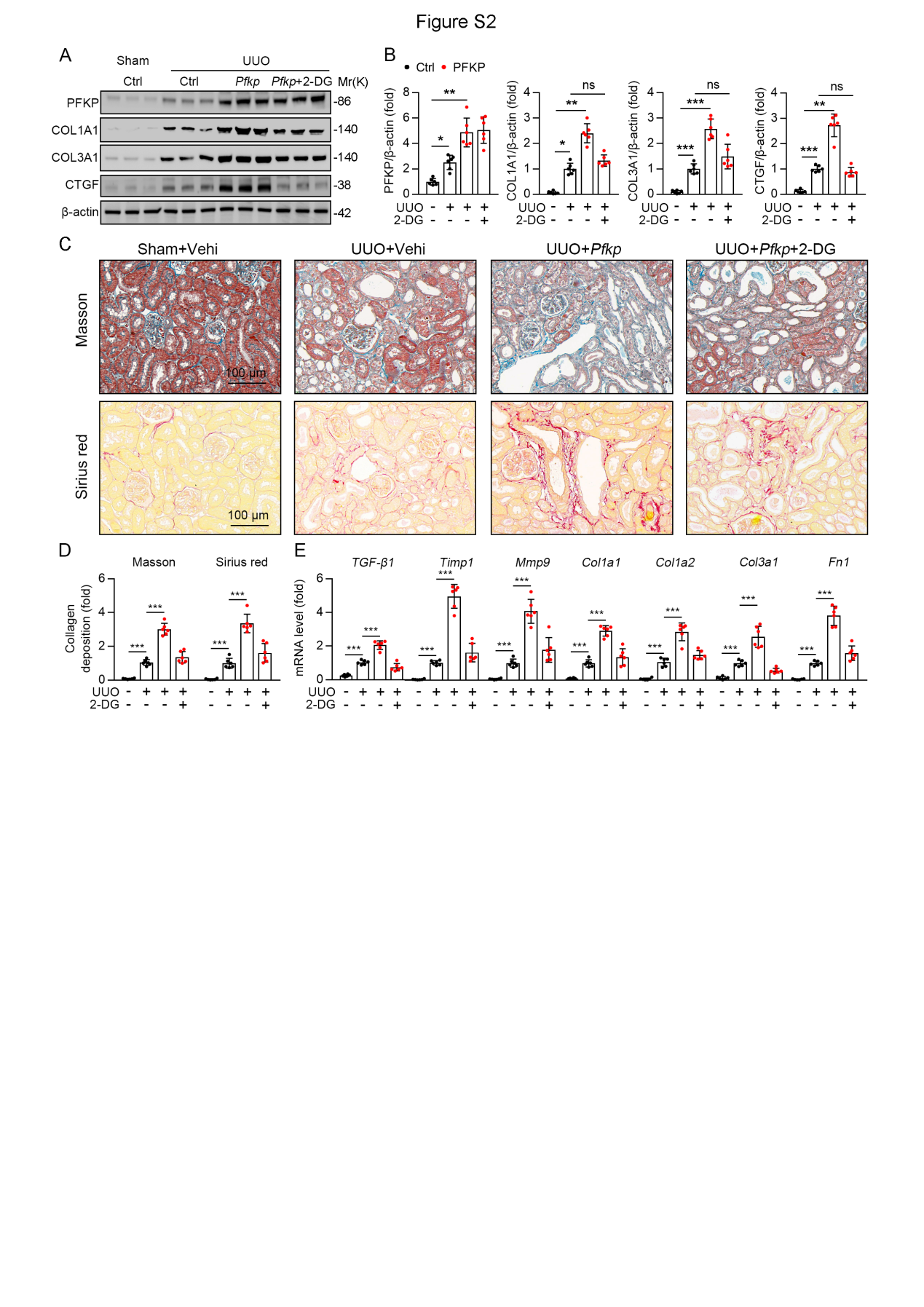
of ten randomly chosen high-power fields (original magnification × 200) in mice kidney sections, n = 6. Right panel: The mRNA level of Nagl and KIM-1 were shown, n = 6. Data are shown as means ± SD; **p* < 0.05, ***p* < 0.01, ****p* < 0.001.

**Figure S2. PFKP promoted renal fibrosis in UUO mice dependent on glycolysis. A.** Immunoblotting showed that the protein levels of *PFKP* and ECM proteins (COL1A1, COL3A1, and CTGF) in UUO mice group and sham group. **B.** The quantitative results of panel A are shown, n = 6 (β-actin was used as the loading control). **C.** Masson staining and Sirius red staining showed that severity of renal fibrosis in UUO mice group and sham group with or without ISO treatment, 3 fields per mice at 200 × magnification, n = 6. **D**. For masson’s trichrome staining and Sirius red staining was quantified in the kidney sections in 3 fields per mice at ×100 magnification, n = 6. **E.** The mRNA expression levels of *Tgf-β1, Timp1, Mmp9, Col1α1, Col1a2, Col3α1, and Fn1* in the renal tissues of mice, n = 6. Data are shown as means ± SD; **p* < 0.05, ***p* < 0.01, ****p* < 0.001.

**Table S1. The sequences of primers for qPCR analysis**

| Gene | Forward Primer (5’→3’) | Reverse Primer (5’→3’) |
| --- | --- | --- |
| *mus-Tgf-β1*  (ID:21803) | CTCCCGTGGCTTCTAGTGC | GCCTTAGTTTGGACAGGATCTG |
| *mus-Fn1*  (ID:14268 ) | ATGTGGACCCCTCCTGATAGT | GCCCAGTGATTTCAGCAAAGG |
| *mus-Col1a1*  (ID:12842 ) | GCTCCTCTTAGGGGCCACT | CCACGTCTCACCATTGGGG |
| *mus-Col1a2*  (ID: 12843) | GTAACTTCGTGCCTAGCAACA | CCTTTGTCAGAATACTGAGCAGC |
| *mus-Col3a1*  (ID: 12825) | CTGTAACATGGAAACTGGGGAAA | CCATAGCTGAACTGAAAACCACC |
| *mus-Timp1*  (ID: 21857) | GCAACTCGGACCTGGTCATAA | CGGCCCGTGATGAGAAACT |
| *mus-Mmp9*  (ID: 17395) | CTGGACAGCCAGACACTAAAG | CTCGCGGCAAGTCTTCAGAG |
| *mus-Ngal*  (ID: 16819) | TGGCCCTGAGTGTCATGTG | CTCTTGTAGCTCATAGATGGTGC |
| *mus Kim-1*  (ID: 171283) | ACATATCGTGGAATCACAACGAC | ACAAGCAGAAGATGGGCATTG |
| *mus-Serpine1*  (ID:18787 ) | TTCAGCCCTTGCTTGCCTC | ACACTTTTACTCCGAAGTCGGT |
| *mus-α-SMA (ID: 11475)* | CCCAACTGGGACCACATGG | TACATGCGGGGGACATTGAAG |
| *homo-PFKP*  ( ID: 5214) | GACCTTCGTTCTGGAGGTGAT | CACGGTTCTCCGAGAGTTTG |

**Table S2 PCR primers used for construction of *PFKP* promoters**

| Gene | Forward Primer (5’→3’) | Reverse Primer (5’→3’) |
| --- | --- | --- |
| -3048 Luc | (CGGGGTACC)GCCTGTCTGAACTCCTGGTC | (TCCCCCGGG)GACGGCAGCGTTCATACCT |
| -2142 Luc | (CGGGGTACC)AGGAGATAAGGGGCATTTGG | (TCCCCCGGG)GACGGCAGCGTTCATACCT |
| -1234 Luc | (CGGGGTACC)AGGTCAGGCAGAGCGTACAG | (TCCCCCGGG)GACGGCAGCGTTCATACCT |
| -158 Luc | (CGGGGTACC)GGTCCCCATTGCCTGCTG | (TCCCCCGGG)GACGGCAGCGTTCATACCT |
