# Supplementary material for: Inhibition of PFKP in renal tubular epithelial cell restrains TGF-β induced glycolysis and renal fibrosis
Source: Cell Death Dis. 2023 Dec 12;14(12):816. doi: 10.1038/s41419-023-06347-1 (PMC10716164; doi:10.1038/s41419-023-06347-1)

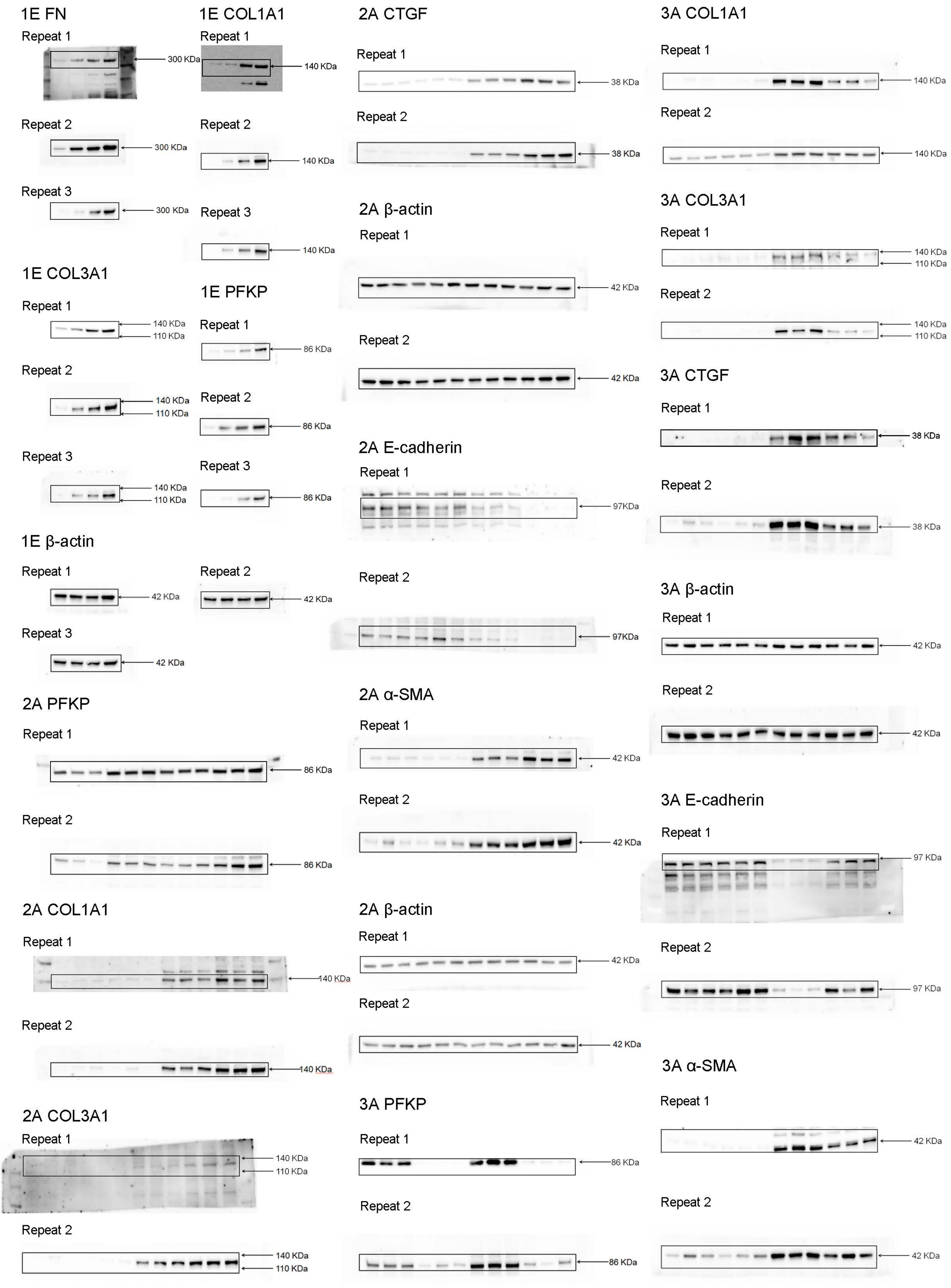
1E FN
Repeat 1
300 KDa
Repeat 2
300 KDa
Repeat 3
300 KDa
1E COL1A1
Repeat 1
140 KDa
Repeat 2
140 KDa
Repeat 3
140 KDa
1E COL3A1
Repeat 1
140 KDa
110 KDa
Repeat 2
140 KDa
110 KDa
Repeat 3
140 KDa
110 KDa
1E PFKP
Repeat 1
86 KDa
Repeat 2
86 KDa
Repeat 3
86 KDa
1E β-actin
Repeat 1
42 KDa
Repeat 2
42 KDa
Repeat 3
42 KDa
2A PFKP
Repeat 1
86 KDa
Repeat 2
86 KDa
2A COL1A1
Repeat 1
140 KDa
Repeat 2
140 KDa
2A COL3A1
Repeat 1
140 KDa
110 KDa
Repeat 2
140 KDa
110 KDa
2A CTGF
Repeat 1
38 KDa
Repeat 2
38 KDa
2A β-actin
Repeat 1
42 KDa
Repeat 2
42 KDa
2A E-cadherin
Repeat 1
97KDa
Repeat 2
97KDa
2A α-SMA
Repeat 1
42 KDa
Repeat 2
42 KDa
2A β-actin
Repeat 1
42 KDa
Repeat 2
42 KDa
3A PFKP
Repeat 1
86 KDa
Repeat 2
86 KDa
3A COL1A1
Repeat 1
140 KDa
Repeat 2
140 KDa
3A COL3A1
Repeat 1
140 KDa
110 KDa
Repeat 2
140 KDa
110 KDa
3A CTGF
Repeat 1
38 KDa
Repeat 2
38 KDa
3A β-actin
Repeat 1
42 KDa
Repeat 2
42 KDa
3A E-cadherin
Repeat 1
97 KDa
Repeat 2
97 KDa
3A α-SMA
Repeat 1
42 KDa
Repeat 2
42 KDa

3A β-actin

Repeat 1

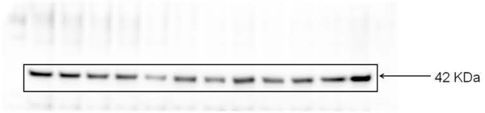

Repeat 2

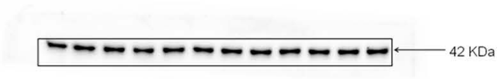

4A p-LDHA

Repeat 1

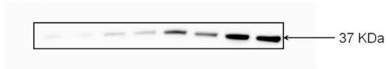

Repeat 2

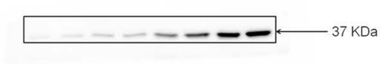

Repeat 3

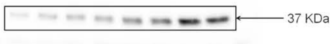

4A LDHA

Repeat 1

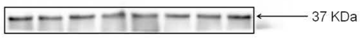

Repeat 2

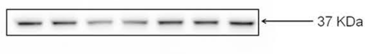

Repeat 3

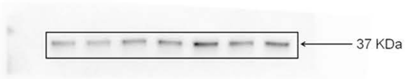

4A HIF-1α

Repeat 1

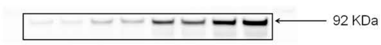

Repeat 2

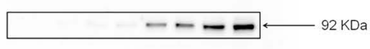

Repeat 3

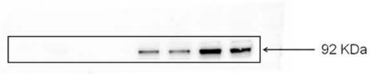

4A HEK2

Repeat 1

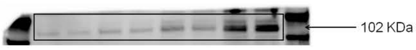

Repeat 2

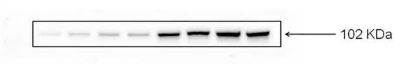

Repeat 3

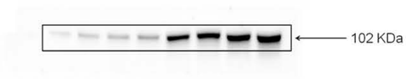

4A p-PKM2

Repeat 1

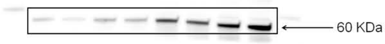

Repeat 2

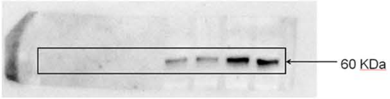

Repeat 3

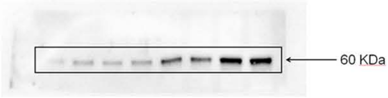

4A PKM2

Repeat 1

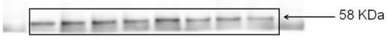

Repeat 2

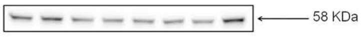

Repeat 3

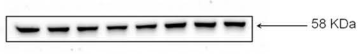

4A β-actin

Repeat 1

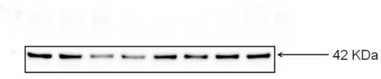

Repeat 2

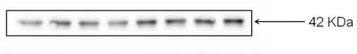

Repeat 3

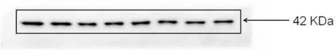

4C p-LDHA

Repeat 1

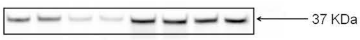

Repeat 2

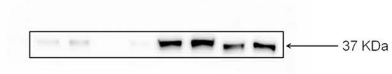

Repeat 3

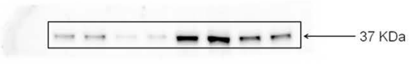

4C LDHA

Repeat 1

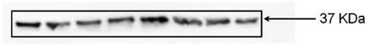

Repeat 2

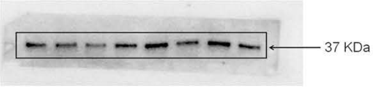

Repeat 3

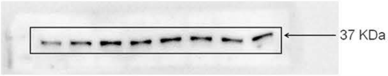

4C HIF-1α

Repeat 1

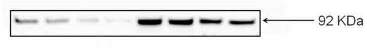

Repeat 2

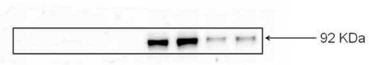

Repeat 3

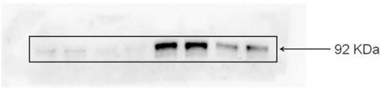

4C HEK2

Repeat 1

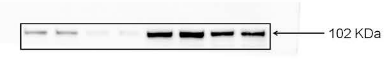

Repeat 2

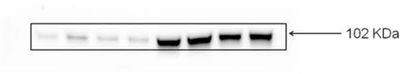

Repeat 3

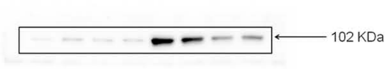

4C p-PKM2

Repeat 1

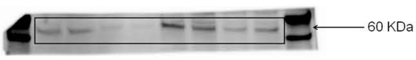

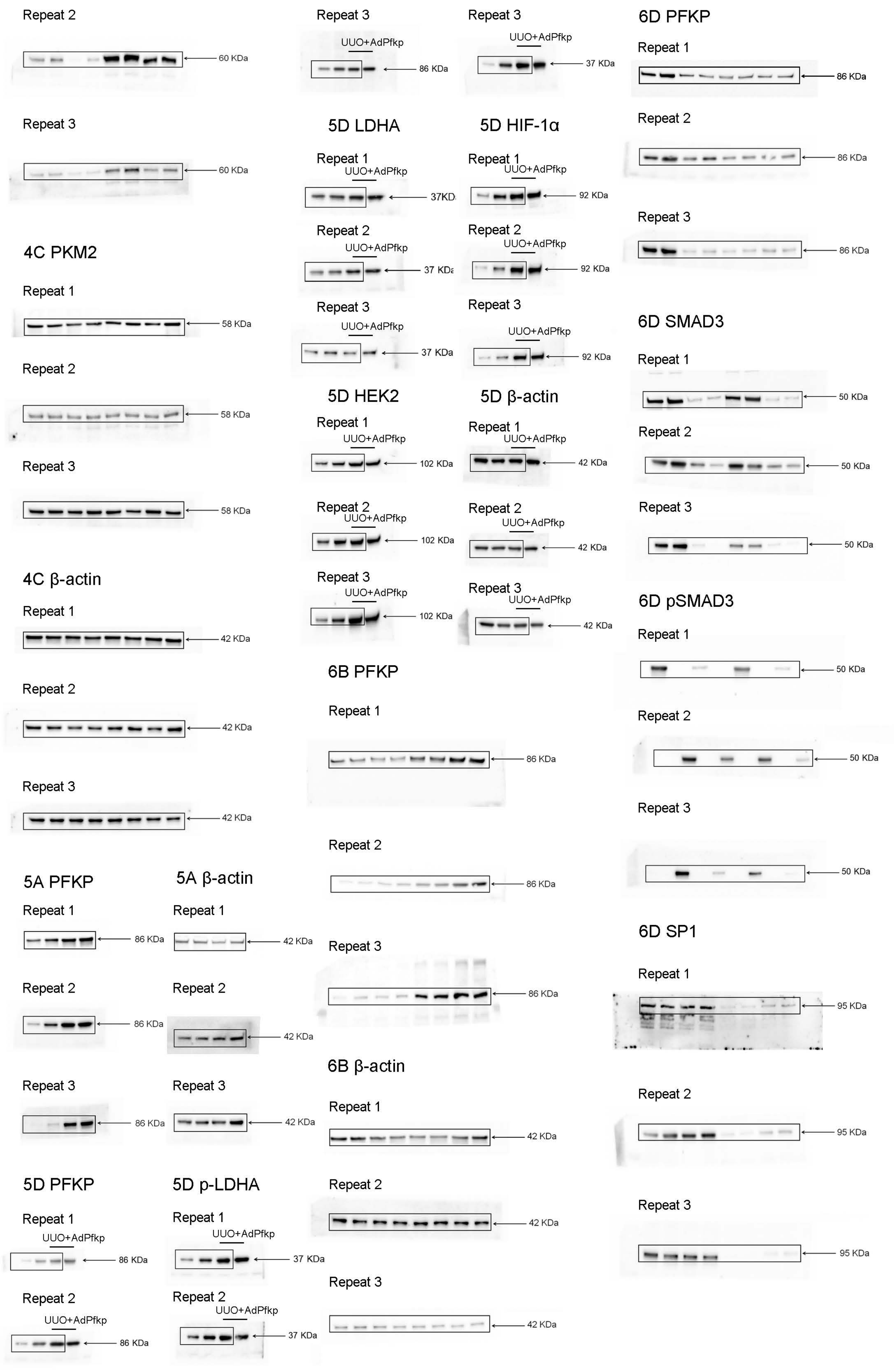
Repeat 2
60 KDa
Repeat 3
60 KDa
4C PKM2
Repeat 1
58 KDa
Repeat 2
58 KDa
Repeat 3
58 KDa
4C β-actin
Repeat 1
42 KDa
Repeat 2
42 KDa
Repeat 3
42 KDa
5A PFKP
Repeat 1
86 KDa
Repeat 2
86 KDa
Repeat 3
86 KDa
5A β-actin
Repeat 1
42 KDa
Repeat 2
42 KDa
Repeat 3
42 KDa
5D PFKP
Repeat 1
UUO+AdPfkp
86 KDa
Repeat 2
UUO+AdPfkp
86 KDa
5D p-LDHA
Repeat 1
UUO+AdPfkp
37 KDa
Repeat 2
UUO+AdPfkp
37 KDa
Repeat 3
UUO+AdPfkp
86 KDa
Repeat 3
UUO+AdPfkp
37 KDa
5D LDHA
Repeat 1
UUO+AdPfkp
37KD
Repeat 2
UUO+AdPfkp
37 KD
Repeat 3
UUO+AdPfkp
37 KDa
5D HIF-1α
Repeat 1
UUO+AdPfkp
92 KDa
Repeat 2
UUO+AdPfkp
92 KDa
Repeat 3
UUO+AdPfkp
92 KDa
5D HEK2
Repeat 1
UUO+AdPfkp
102 KDa
Repeat 2
UUO+AdPfkp
102 KDa
Repeat 3
UUO+AdPfkp
102 KDa
5D β-actin
Repeat 1
UUO+AdPfkp
42 KDa
Repeat 2
UUO+AdPfkp
42 KDa
Repeat 3
UUO+AdPfkp
42 KDa
6B PFKP
Repeat 1
86 KDa
Repeat 2
86 KDa
Repeat 3
86 KDa
6B β-actin
Repeat 1
42 KDa
Repeat 2
42 KDa
Repeat 3
42 KDa
6D PFKP
Repeat 1
86 KDa
Repeat 2
86 KDa
Repeat 3
86 KDa
6D SMAD3
Repeat 1
50 KDa
Repeat 2
50 KDa
Repeat 3
50 KDa
6D pSMAD3
Repeat 1
50 KDa
Repeat 2
50 KDa
Repeat 3
50 KDa
6D SP1
Repeat 1
95 KDa
Repeat 2
95 KDa
Repeat 3
95 KDa

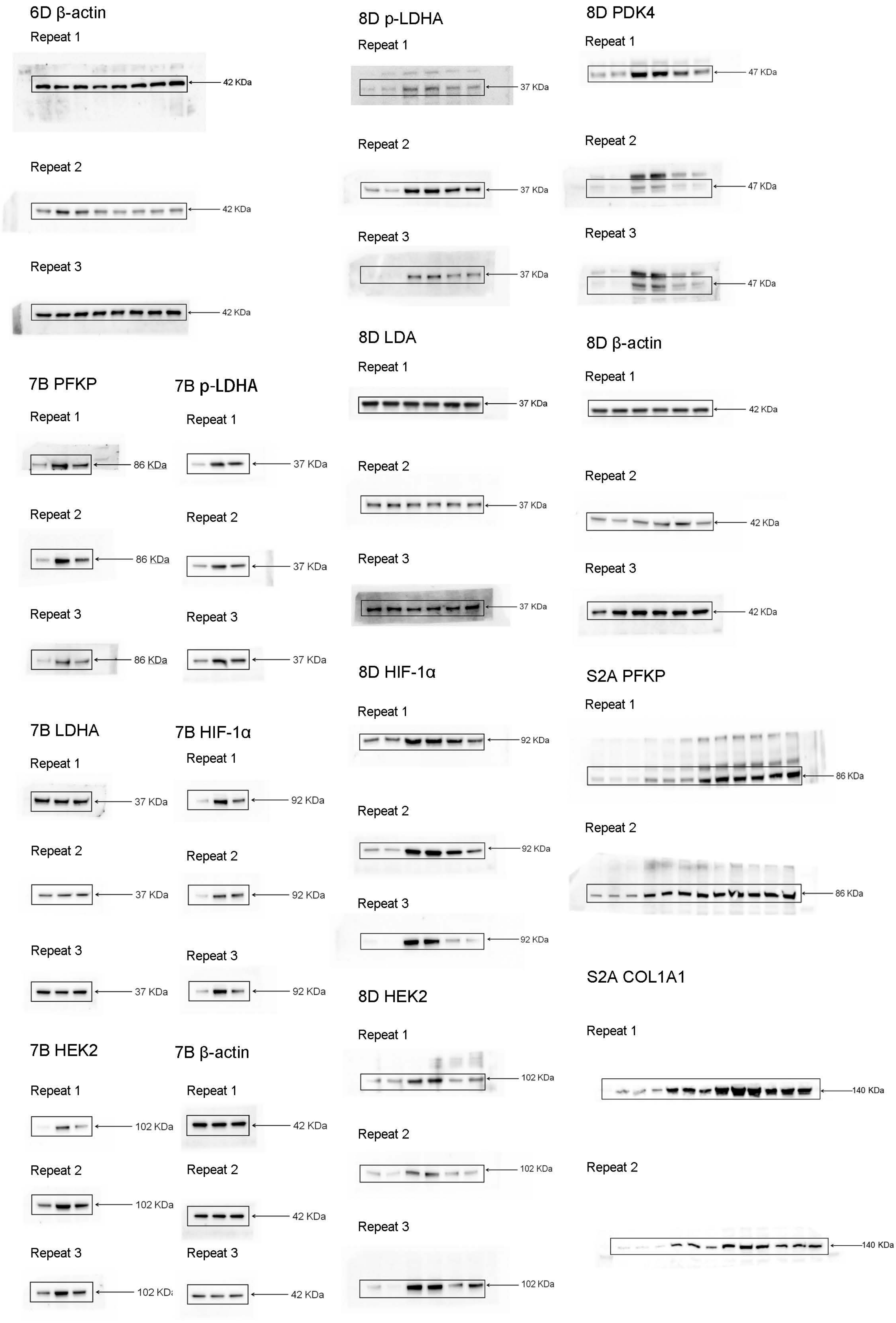

6D β-actin
Repeat 1
42 KDa
Repeat 2
42 KDa
Repeat 3
42 KDa
7B PFKP
Repeat 1
86 KDa
Repeat 2
86 KDa
Repeat 3
86 KDa
7B p-LDHA
Repeat 1
37 KDa
Repeat 2
37 KDa
Repeat 3
37 KDa
7B LDHA
Repeat 1
37 KDa
Repeat 2
37 KDa
Repeat 3
37 KDa
7B HIF-1α
Repeat 1
92 KDa
Repeat 2
92 KDa
Repeat 3
92 KDa
7B HEK2
Repeat 1
102 KDa
Repeat 2
102 KDa
Repeat 3
102 KD
7B β-actin
Repeat 1
42 KDa
Repeat 2
42 KDa
Repeat 3
42 KDa
8D p-LDHA
Repeat 1
37 KDa
Repeat 2
37 KDa
Repeat 3
37 KDa
8D LDA
Repeat 1
37 KDa
Repeat 2
37 KDa
Repeat 3
37 KDa
8D HIF-1α
Repeat 1
92 KDa
Repeat 2
92 KDa
Repeat 3
92 KDa
8D HEK2
Repeat 1
102 KDa
Repeat 2
102 KDa
Repeat 3
102 KDa
8D PDK4
Repeat 1
47 KDa
Repeat 2
47 KDa
Repeat 3
47 KDa
8D β-actin
Repeat 1
42 KDa
Repeat 2
42 KDa
Repeat 3
42 KDa
S2A PFKP
Repeat 1
86 KDa
Repeat 2
86 KDa
S2A COL1A1
Repeat 1
140 KDa
Repeat 2
140 KDa

S2A COL3A1

Repeat 1

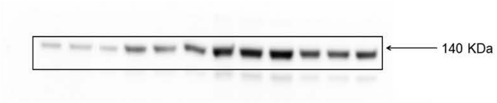

Repeat 2

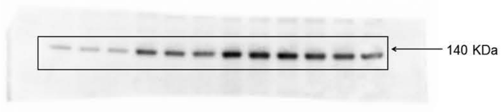

S2A CTGF

Repeat 1

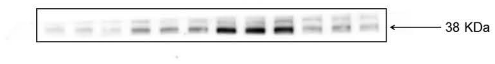

Repeat 2

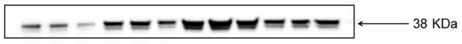

S2A β-actin

Repeat 1

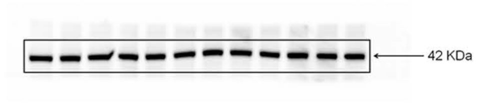

Repeat 2

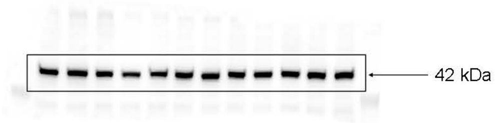

Supplement: Supplementary file 2 — Original Data [file 41419_2023_6347_MOESM2_ESM.pdf]
